# Multishell Silver Indium Selenide-Based Quantum Dots and Their Poly(methyl methacrylate) Composites for Application in Red-Light-Emitting Diodes

**DOI:** 10.1021/acsami.4c06433

**Published:** 2024-07-05

**Authors:** Lorenzo Branzi, Jinming Liang, Garret Dee, Aoife Kavanagh, Yurii K. Gun’ko

**Affiliations:** School of Chemistry, CRANN and AMBER Research Centres, Trinity College Dublin, College Green, Dublin 2 D02 PN40, Ireland

**Keywords:** silver indium selenide, quantum dots, ternary quantum dots, pmma, led

## Abstract

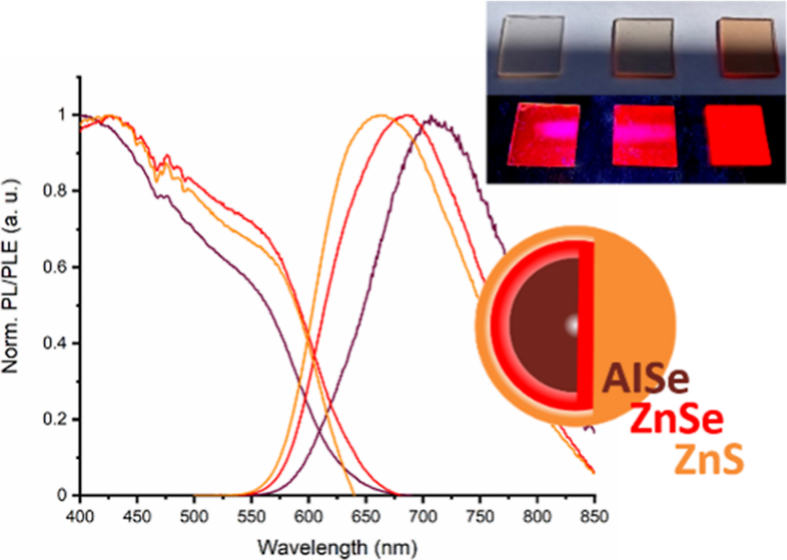

In this work, the production of novel multishell silver indium selenide quantum dots (QDs) shelled with zinc selenide and zinc sulfide through a multistep synthesis precisely designed to develop high-quality red-emitting QDs is explored. The formation of the multishell nanoheterostructure significantly improves the photoluminescence quantum yield of the nanocrystals from 3% observed for the silver indium selenide core to 27 and 46% after the deposition of the zinc selenide and zinc sulfide layers, respectively. Moreover, the incorporation of the multishelled QDs in a poly(methyl methacrylate) (PMMA) matrix via in situ radical polymerization is investigated, and the role of thiol ligand passivation is proven to be fundamental for the stabilization of the QDs during the polymerization step, preventing their decomposition and the relative luminescence quenching. In particular, the role of interface chemistry is investigated by considering both surface passivation by inorganic zinc chalcogenide layers, which allows us to improve the optical properties, and organic thiol ligand passivation, which is fundamental to ensuring the chemical stability of the nanocrystals during in situ radical polymerization. In this way, it is possible to produce silver-indium selenide QD-PMMA composites that exhibit bright red luminescence and high transparency, making them promising for potential applications in photonics. Finally, it is demonstrated that the new silver indium selenide QD-PMMA composites can serve as an efficient color conversion layer for the production of red light-emitting diodes.

## 1 Introduction

Ternary quantum dots (QDs) of the I–III–VI family, such as silver and copper indium sulfides and selenides, are becoming of large interest as potentially more biocompatible alternatives to II–VI and IV–VI binary QDs.^1^ Moreover, the characteristic optical properties of I–III–VI QDs, such as large Stokes shift and broad luminescence spectra, are of high interest for application in luminescent solar concentrators and white light-emitting diodes (LEDs).^2^ These properties are strictly related to the exciton recombination mechanisms that characterize I–III–VI ternary QD photoluminescence, such as the involvement of mid-gap states in the radiative recombination as well as the presence of a strong exciton–phonon coupling.^3,4^

Among the rich family of I–III–VI QDs, the scientific community has been mainly focused on the sulfide systems such as silver indium sulfide and copper indium sulfide. The less investigated class of selenides is of large interest due to their smaller band gap, which allows for obtaining emission in the red and near-infrared spectral range, which is promising for several applications such as biological imaging. Silver indium selenide is a direct band gap semiconductor with an optical band gap around 1.19 eV observed for the bulk material.^5−7^ In respect to the more popular sulfide compounds, fewer synthetic approaches are available in the literature for the production of silver indium selenide QDs. This has been mainly related to the different chemical reactivity between the cationic and selenide precursors, which gives poor control over the particle morphology, structure, and chemical compositions, leading to a product characterized by very broad optical features.^8^ Some protocols on the aqueous synthesis of silver indium selenides have been recently reported; an example is the coprecipitation reaction of silver nitrate and indium chloride using a freshly prepared sodium selenosulfate (NaSeSO_3_) solution as a selenium source in the presence of glutathione as a stabilizer.^9^ Under reflux conditions, sodium selenosulfate decomposes, releasing selenide anions in situ. In this way, it was possible to prevent the oxidation of Se^0^ even when operating in quasi “inert” conditions. Another coprecipitation method was reported by Kang et al.^10^ for large-scale production of silver indium selenide QDs using thioglycolic acid and gelatin as ligands and a solution as NaHSe as the selenium source. These aqueous methods are of large interest for their application in the biological system; however, QDs are usually characterized by poorer optical properties with a lower photoluminescence quantum yield (PLQY) and broad size distribution. For this reason, a large amount of effort has been spent on hot-injection methods for the production of silver indium selenide QDs in the organic phase. Among these, different selenium precursors have been proven to be useful for the production of high-quality silver indium selenide QDs. Allen et al.^11^ investigated the use of bis(trimethylsilyl)selenide in tri-*n*-octylphosphine for the production of copper and silver indium selenide QDs using silver and indium iodide as cation precursors. Yarema et al.^12^ used a selenium solution in tri-*n*-octylphosphine as a selenium precursor solution combined with lithium bis(trimethyl)silyl amide to increase the reactivity of silver and indium iodide by precipitating lithium iodide and forming metal amide intermediate species. Through this procedure, it was possible to achieve unprecedented control over QD size and chemical composition. Yao et al.^13^ investigated the use of selenium solubilized in oleylamine in the presence of dodecanethiol. This strategy allows us to exploit the oxidation of alkylthiols to the corresponding disulfide, forming oleylamine–selenium complexes soluble at room temperature.^14^ This approach has been successfully used to produce high-quality selenide-based QDs at relatively mild temperatures. Post-synthetic passivation of the QDs with an inorganic shell layer is usually employed to improve the optical properties. For this purpose, ZnSe and ZnS are widely used to produce Core/Shell QDs via epitaxial deposition on a preformed QD. ZnSe and ZnS are of particular interest due to their wide band gap and similar crystallographic structure to the silver indium selenide core, fundamental criteria to confine the photogenerated excitons in the core material while minimizing interface defects.^8,12,15^ Thick shells have been proven to successfully boost the QDs’ optical properties and stability; however, a large blue shift is typically observed in this condition due to the migration of zinc ions in the core layer producing yellow-green-emitting QDs.^12,16−19^ For this reason, the preparation of high-quality red-emitting ternary QDs for photonics applications is particularly challenging.

The large interest in the application of QDs in photonic devices has stimulated several researchers to investigate their incorporation in polymeric matrixes for the production of multiple devices, including luminescence solar concentrators,^20−25^ scintillators,^26^ and color conversion layers for (LEDs).^13,27^ In particular, bulk radical polymerization is of high interest for its versatility and potential industrial application.^21^ However, the harsh reaction conditions are often incompatible with the presence of the nanoparticles, and a significant reduction in the QD photoluminescence is commonly related to the reaction with radicals and combined with a poor homogeneity of the composite due to phase separation.^28−30^ A large focus on in situ polymerization in the presence of nanoparticles has been dedicated to Cd-based QDs and ZnS nanoparticles in poly(methyl methacrylate) (PMMA) or octadecyl-*p*-vinyl benzyl dimethyl ammonium chloride (OVDAC).^21,28,31,32^ Only recently, the incorporation of ternary I–III–VI QDs has been reported; in particular, Dhamo et al.^22^ investigated the incorporation of core/shell silver indium sulfide/ZnS QDs in the polylauroyl methacrylate matrix produced by photopolymerization under UV light. Similarly, Koch et al.^23^ used copper indium sulfide/ZnS QDs coated with the polymeric ligand to produce a PMMA composite via polymerization of a PMMA/MMA mixture.

Here, we present the synthesis and optical properties of silver indium selenide QDs shelled by the deposition of a first zinc selenide layer (AISe/ZnSe) and a second zinc sulfide layer (AISe/ZnSe/ZnS). The deposition of the shell layer has proven to efficiently raise the PLQY from 3% for the AISe core to 27% and finally 46% for the ZnSe and ZnSe/ZnS layers, respectively, while preserving a red emission. Moreover, we investigated the incorporation of AISe/ZnSe/ZnS QDs in the PMMA matrix via a simple in situ polymerization method. We observed that unlike other QDs, silver indium selenide QDs are highly sensitive to the reaction conditions during the polymerization reaction, and the radical species act as oxidizers to decompose the nanoparticles with the production of Se^0^. The production of a high-quality red-mitting QDs-PMMA composite was possible by optimizing the interface chemistry. In particular, combining the surface passivation provided by the inorganic zinc chalcogenide shells, which improve the QDs’ optical properties, with appropriate ligand stabilizers such as thiols, which are fundamental to preventing the QDs oxidation during the polymerization step. Finally, the new silver indium selenide QD-PMMA composite was investigated for application as a color conversion layer for LEDs for the production of red LEDs.

## 2 Results and Discussion

### 2.1 Multishell Silver Indium Selenide QD Synthesis

**Figure 1:**
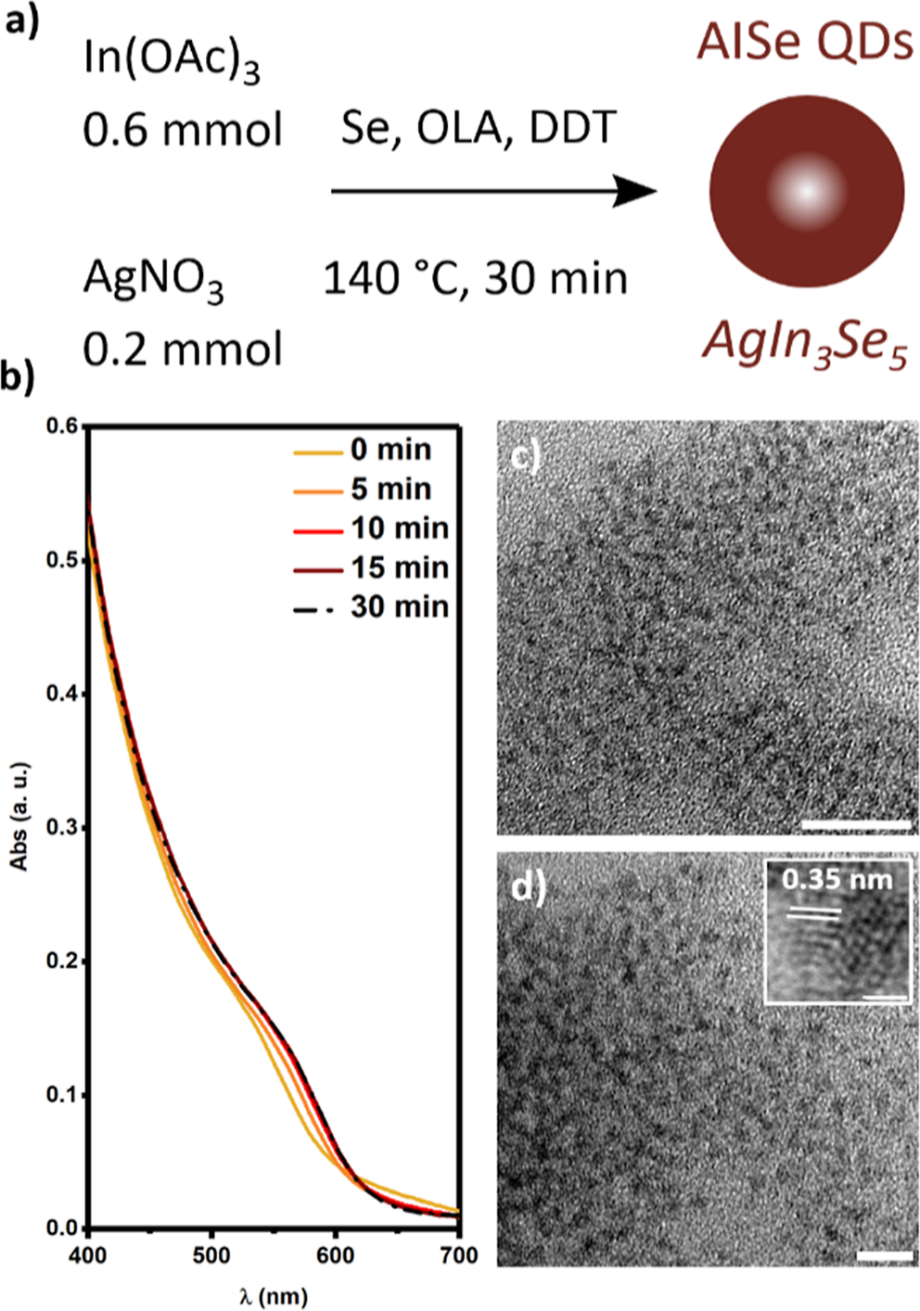
(a) Representation of the synthetic scheme adopted for the preparation of AISe QDs. (b) UV/vis absorption spectra of the reaction mixture collected at different times after the injection of the selenium precursor solution. (c, d) TEM images of AISe QDs; the scale bars correspond to *c* = 20 nm, *d* = 10 nm, and *d* inset 1 nm.

I–III–VI silver indium selenides (AISe) QDs were synthesized by the hot injection method using a relatively low reaction temperature (Figure 1a). We focused our attention on the indium-rich AgIn_3_Se_5_ composition; this is a known vacancy-ordered compound which presents superior optical properties (in particular PLQY) with respect to AgInSe_2_, and for this reason, it is more appealing for applications in photonics.^12^ The selenium precursor solution was prepared by the solubilization of elemental selenium powder in oleylamine in the presence of a stoichiometric amount of 1-dodecanethiol. The addition of 1-dodecanethiol allowed the selenium powder to solubilize at room temperature, producing disulfides.^14^ The selenium precursor solution was rapidly added to the reaction mixture containing silver and indium salts solubilized in a 1-octadecene, 1-dodecanethiol, and oleic acid (OA) mixture at 140 °C. A molar ratio of 1:3 between silver nitrate and indium acetate was used to promote the formation of QDs with the desired stoichiometry. The relatively mild reaction temperature is crucial to prevent the decomposition of 1-dodecanethiol, which would produce undesired sulfide anions in the reaction mixture.^33^ The injection of the selenium solutions causes the immediate formation of nanocrystals; the UV–vis absorption spectra in Figure 1b show the absorption of the reaction mixture collected at different reaction times. A red shift of the absorption band from 560 to 580 nm is observed in the first 15 min of the reaction and can be related to the increase in the nanocrystal size. A longer reaction time does not affect the absorption spectra, suggesting that the nanocrystal growth is terminated in the first 15 min. Transmission electron microscopy (TEM) micrographs of the AISe QDs (Figure 1c,d) show the presence of nanocrystals with an average size distribution of 2.4 ± 0.5 nm. High-resolution transmission electron microscopy (HR-TEM) analysis reveals a *d*-spacing of 0.35 nm that is related to the (112) planes of tetragonal AgInSe_2_. Energy dispersive X-ray spectroscopy (EDS) reveals the chemical composition of the AISe QDs, and the analysis shows a silver-to-indium atomic ratio of 1:2.99, which confirms the successful production of the indium-rich AgIn_3_Se_5_ QDs.

**Figure 2:**
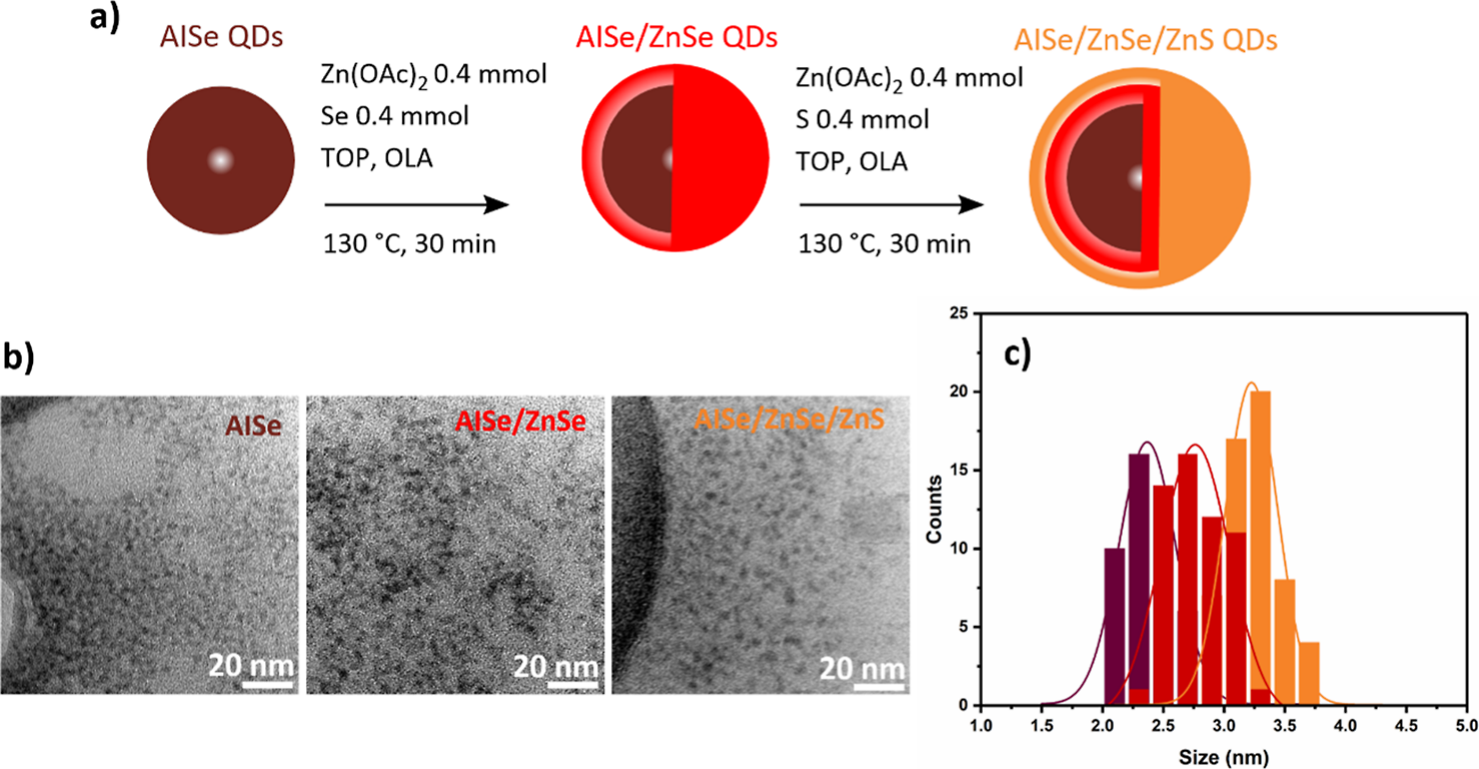
(a) Representation of the synthetic scheme used for the deposition of ZnSe and ZnS shell layers. (b) TEM images of AISe, AISe/ZnSe, and AISe/ZnSe/ZnS QDs. (c) Size distribution histogram of AISe (brown), AISe/ZnSe (red), and AISe/ZnSe/ZnS QDs (orange).

To improve the QD optical properties, AISe QDs were passivated by the deposition of ZnSe and ZnS layers following the reaction scheme represented in Figure 2a. The multiple shelling steps allowed for the production of AISe/ZnSe and AISe/ZnSe/ZnS QDs via separate additions of ZnSe and ZnS precursor solutions. These solutions are based on the solubilization of selenium and sulfur powders as TOP-chalcogenide species, and the solubilization of zinc acetate is favored by the addition of a hard Lewis base ligand such as oleylamine. The ZnSe layer is particularly beneficial to passivate the AISe QD surface with a larger band gap semiconductor layer while minimizing the lattice mismatch with respect to ZnS.^12,34^ The second deposition of a ZnS layer allows us to further insulate QDs with an even wider band gap semiconductor and enhances the chemical stability toward oxidation reactions. The final particles showed high stability under an environmental atmosphere and can be stored in air (closed vials) for months without any significant quenching of their luminescence. The TEM images shown in Figure 2b present the morphology of QDs isolated at different steps of the shelling process, along with the corresponding size distributions shown in Figure 2c. The average particle size evidences the growth of the QDs from the original AISe core size of 2.4 ± 0.5–2.8 ± 0.5 nm for the AISe/ZnSe core–shell QDs and 3.4 ± 0.4 nm for the AISe/ZnSe/ZnS core/shell/shell QDs. The small variation in particle size suggests that the process gives to the deposition of a thin layer of ZnSe or ZnS approximately around the monolayer range (∼0.31 nm); similar observations were reported by other authors.^8,10^

**Figure 3:**
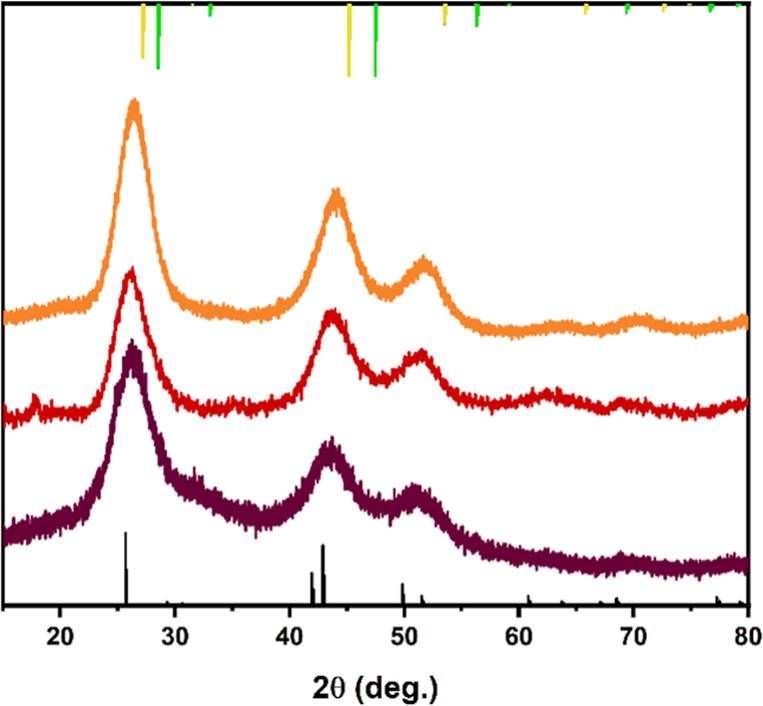
X-ray diffraction analysis (XRD) pattern of AISe (brown), AISe/ZnSe (red), and AISe/ZNSe/ZnS (orange) QDs. Reference pattern: AgInSe_2_ chalcopyrite phase ICSD28751 (black), ZnSe sphalerite phase ICSD77091 (yellow), and ZnS sphalerite phase ICSD77090 (green).

XRD and EDS analyses have been used to further clarify the modification of the QDs during the process. The XRD analysis (Figure 3) shows that all the systems present the typical diffraction pattern expected for the AgInSe_2_ tetragonal chalcopyrite phase (space group *I*4̅2*d*), in particular the main diffractions observed in the AISe QD core are centered at 26.2, 43.5, and 51.1° 2θ and can be related to the (112), (201, 220), and (312,116) planes, which is in good agreement with the reference pattern for the AgInSe_2_ chalcopyrite phase.^11,35^ Similar observations regarding the crystallographic structure of off-stoichiometric I–III–VI QDs were reported by other authors.^13,36,37^ The shelling steps, first with ZnSe and followed by ZnS, caused a slight shift of the diffraction peaks toward higher angles, which is associated with the incorporation of ions of smaller radii like zinc and sulfur.^24^ In particular, the (201, 220) diffraction is observed at 43.5, 43.8, and 44.1° 2θ for AISe, AISe/ZnSe, and AISe/ZnSe/ZnS QDs, respectively. The (312,116) diffraction shows a similar trend with 51.1, 51.6, and 52.0° 2θ for AISe, AISe/ZnSe, and AISe/ZnSe/ZnS, respectively. This observation is in line with the epitaxial growth of ZnSe and ZnS layers with a sphalerite (zincblende) structure.^38^

The EDS analysis (Figure S1) reveals the variation of the QD chemical composition; in particular, the AISe QD core shows an atomic ratio of 1:2.99:4.8 for silver, indium, and selenium, respectively, which is in line with the formation of the desired AgIn_3_Se_5_ core and suggests the formation of silver vacancies and an indium substitute for silver defects in the chalcopyrite nanocrystals.^13,36^ Interestingly, the silver-to-indium ratio is preserved in the core–shell systems with 1:3.04 and 1:2.90 observed for AISe/ZnSe and AISe/ZnSe/ZnS QDs, respectively, indicating that in the synthetic condition used to deposit the shell layers, the AgIn_3_Se_5_ core remains mainly unaffected and alloying via cation exchange is minimized. This is further confirmed by the optical characterizations. This is probably due to the mild reaction conditions chosen to deposit the shell layers, and it is particularly beneficial for the production of red or near-infrared-emissive QDs since zinc alloying can cause variation in the composition of the QDs and a large blue-shift of the emission maximum.^16,38^ The deposition of ZnSe and ZnS layers can be monitored by considering the zinc content, which is extrapolated by the Kα and Lα lines at 8.630 and 1.012 keV, respectively. The silver-to-zinc atomic ratio is 1:0.85 for the AISe/ZnSe QDs and rises to 1:0.93 for AISe/ZnSe/ZnS QDs, confirming the successful deposition of the ZnSe and ZnS layers.

**Figure 4:**
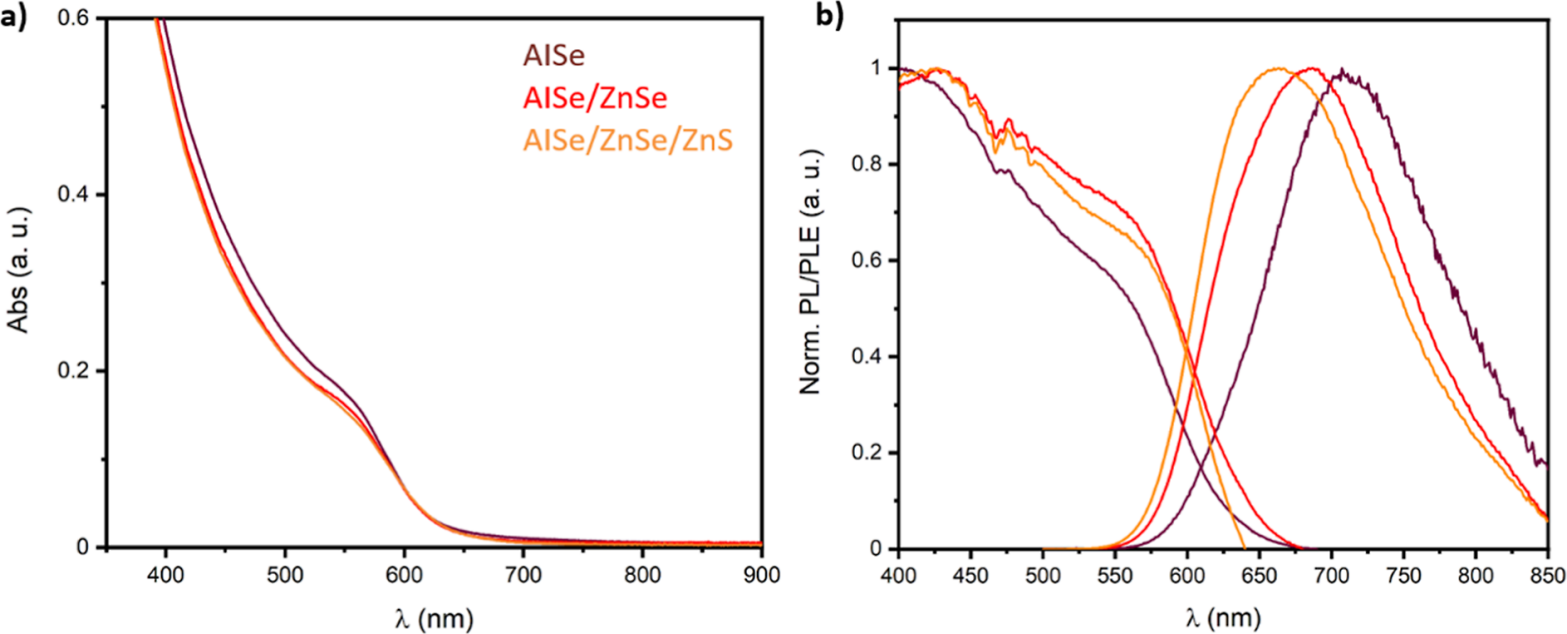
(a) UV–vis and (b) PL/PLE spectra of AISe (brown), AISe/ZnSe (red), and AISe/ZnSe/ZnS (orange) QDs.

The UV/vis spectra (Figure 4a) show similar trends for both the AISe core and the shelled systems. In all cases, the absorption band at 580 nm can be related to the AISe QD core. The energy gap evaluated according to the Tauc Plot (Figure S2) shows an optical band gap around 2.04 eV for both the AISe core and AISe/ZnSe and AISe/ZnSe/ZnS QDs, remarking that the shelling deposition minimally affects the structure of the AgIn_3_Se_5_ core. The larger band gap with respect to the values observed for the bulk semiconductor can be related to the quantum confinement effect, combined with the effect of the chemical composition.^12,39^ In contrast, the photoluminescence properties (Table 1) are dramatically affected by the deposition of the zinc chalcogenide shell layers. The photoluminescence characterization of AISe QDs (Figure 4b) shows a broad emission centered at 710 nm characterized by a full width half-maximum (fwhm) of 141 nm. The broad emission spectra are characteristic of I–III–VI QDs and have been associated with the radiative recombination mechanism involved in these nanocrystals. In particular, several investigations on the photophysics of I–III–VI QDs demonstrated that a donor–acceptor recombination process involving midgap states is responsible for the large Stokes shift. This, along with a strong exciton–phonon coupling gives broad emission spectra.^4,40−43^

**Table 1:** Main Optical Parameters Measured for AISe, AISe/ZnSe, and AISe/ZnSe/ZnS QDs

	λ_em_ (nm)	fwhm (nm)	PLQY	τ_av_ (ns)
AISe	710	141	3	187
AISe/ZnSe	685	149	27	216
AISe/ZnSe/ZnS	670	148	46	252

The photoluminescence excitation spectra show a broad excitation range that covers most of the visible spectrum and starts to decrease at around 580 nm, following the same trend observed in the UV–vis absorption spectra. The emission maximum for the shelled system is blue-shifted with respect to the 710 nm (1.75 eV) emission observed for the AISe QD core to 685 (1.81 eV) and 670 nm (1.85 eV) for AISe/ZnSe and AISe/ZnSe/ZnS QDs, respectively. The fwhm slightly rises after the shelling from 141 nm for the AISe QDs to 149 and 148 nm for AISe/ZnSe and AISe/ZnSe/ZnS QDs, respectively. This behavior is often related to the zinc intercalation in the QD core, causing a widening of the band gap by the formation of alloy nanocrystals.^12,16−19^ However, due to the similarity in UV–vis absorption and PLE spectra among core and shelled systems, a modification of the core composition due to alloying seems unlikely. The blue-shift of the emission spectra can be mainly related to the variation of the interface chemistry due to the deposition of the zinc chalcogenide shells, which significantly affects the surface states that are involved in the more energetic component of the emission spectra.^4^ Moreover, the deposition of the zinc chalcogenide shell layers reduces surface trap states involved in the nonradiative recombination, raising the relative contribution of more energetic components such as surface states.^40^ This difference in the zinc intercalation can be due to the relatively mild reaction temperature used in the shelling procedure as well as to the amount of the zinc chalcogenide precursor injected in each step. The photoluminescence decay data (Figure S3) were fitted with a triexponential model (eq S1), which takes into account the multiple radiative recombination mechanisms that are involved in the photophysics of silver indium selenide QDs. In particular, a recent work by Zacharia et al.^42^ evidenced the presence of three main recombination mechanisms in indium-rich Ag_3_In_5_Se_9_ QDs. The authors assigned these transitions between the minimum of the conduction band to midgap-localized states of self-trapped holes, the maximum of the valence band, and a lower-energy valence band state. In our observations, both AISe QD core and shelled systems show an average lifetime of hundreds of nanoseconds, which is in line with the reported values for silver indium selenide QDs.^12,35^ Moreover, an increase in the average lifetime τ_av_ (eq S2) with the deposition of the shell layers from the AISe core 187–216 and 252 ns for the AISe/ZnSe and AISe/ZnSe/ZnS, respectively, was observed (Table S1). This is related to the suppression of nonradiative surface recombination in surface trap states.^42^ Finally, a dramatic variation is observed in the PLQY (eqs S3 and S4), which increases from 3% as observed for the AISe QDs to 27 and 46% for the AISe/ZnSe and AISe/ZnSe/ZnS QDs in accordance with the variation of the nanocrystal interface, proving the successful passivation of surface defects with Zn chalcogenides shell layers. It is important to remark that the synthetic procedures used to deposit the Zn chalcogenide shell layers were optimized in order to increase the PLQY while preserving an emission in the red spectral region for potential application in red-LEDs. The final PLQY, despite being lower than other red-emitting QDs,^44^ is among the highest values observed for silver indium selenide QDs reported in the literature.^9,11−13,45,46^ A dramatic limitation is imposed by the blue shift of the emission peaks observed with the incorporation of zinc ions in the nanocrystal structure. This phenomenon is well documented in the literature regarding I–III–VI QDs and appears to be especially impactful for the case of selenides.^16−19^ We also explored the deposition of a thicker mixed ZnSe/ZnS shell of the AgIn_3_Se_5_ core (Supporting Information: Thick shell AISe/Zn(SeS) QDs, synthesis, and characterizations) to produce a AISe/Zn(SeS) core–shell QDs (Figure S4). The larger shell improves the PLQY from 3% observed for the AISe core up to 53% for the core–shell QDs, supporting that the larger shell can further increase the PLQY. However, as expected, the deposition of the thicker Zn chalcogenide shell causes a significant blue shift of the luminescence to 610 nm (Figure S5), producing orange emitting QDs.

### 2.2 Silver Indium Selenide-Based QDs-PMMA Composite

**Figure 5:**
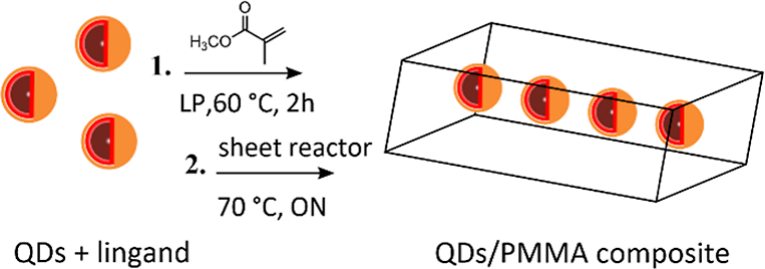
Schematic presentation of the two-step radical polymerization process used to produce AISe/ZnSe/ZnS QDs-PMMA composites.

Since the multishell AISe/ZnSe/ZnS QDs show the most promising optical properties with the highest PLQY (46%) and a red emission (670 nm), this sample has been employed for the preparation of QDs-PMMA composites. Due to its optical clarity and high mechanical strength, PMMA is an ideal matrix for the production of QDs-polymer composites with potential applications in photonics devices.^21,26^ For this purpose, we adopted a two-step process for the production of the composites (Figure 5). In the first prepolymerization step, AISe/ZnSe/ZnS QDs are mixed with the methyl methacrylate monomer and lauroyl peroxide (LP), which is used as a radical initiator, and the solution is heated at 60 °C for 2 h to form a syrup.^47^

**Figure 6:**
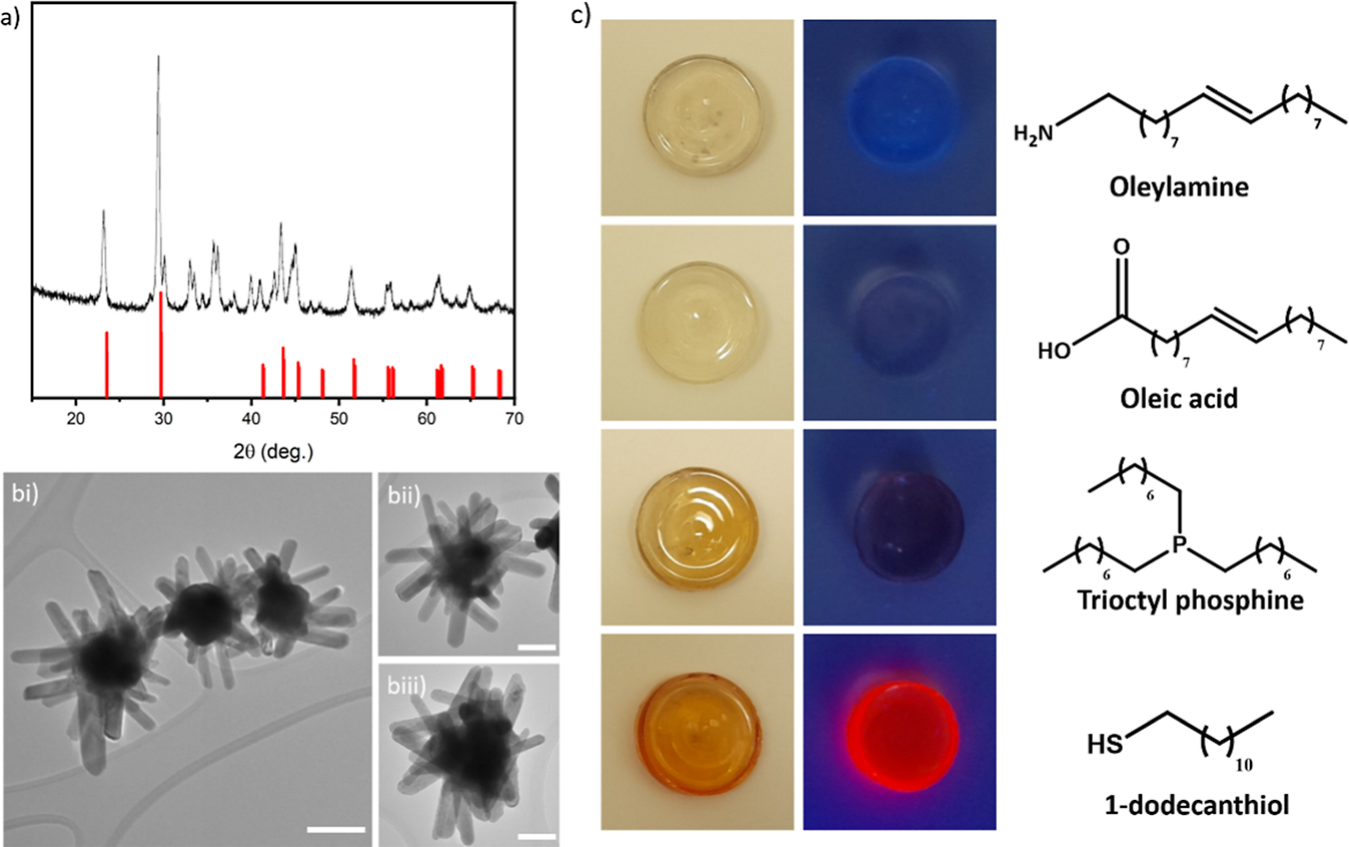
(a) XRD pattern and (b, i–iii) TEM images of the particles isolated during the prepolymerization step. Reference patterns (red): selenium *P*3_1_21 ICSD 40018. Scale bars: (i) = 100 nm and (ii,iii) = 50 nm. (c) AISe/ZnSe/ZnS-PMMA composites prepared in the presence of different ligands under ambient and UV light.

During the second step, the syrup is loaded into a sheet reactor (mold) prepared using two tempered glass sheets separated by a polyvinyl chloride (PVC) gasket, and the polymerization is completed by heating the sheet reactor at 70 °C overnight. We observed that simply adding the AISe/ZnSe/ZnS QD colloidal solution to the reaction mixture causes a rapid quenching of the QD emission during the prepolymerization step. Dramatic QD quenching due to the reaction with initiator radicals has been documented, and a proper strategy to limit QD exposure to the initiator radicals has proven successful for giant CdSe/CdS QDs.^21^ However, even minimizing the contact with the radicals did not prove successful in preventing the luminescence quenching in the case of silver-indium selenide QDs. We have investigated the possible modification to the AISe/ZnSe/ZnS QDs by repeating the prepolymerization in the presence of a large amount of QDs and arresting the process in order to collect the nanoparticles from the reaction mixture via centrifugation. Interestingly, the XRD analysis of the pellet (Figure 6a) shows a dramatic variation of the QD structure, and the main peaks can be related to elemental selenium; other peaks may be related to other byproducts such as salts and coordination complexes. TEM micrographs Figure 6 b(i–iii) show a significant variation in particle morphology during the polymerization reaction, and large hedgehog microcrystals composed of an assembly of rods with a lateral size of around 20 nm and a length of hundreds of nanometers can be observed. We associated this phenomenon with the oxidative activity of the radical species formed during the polymerization reaction, which can oxidize the selenide anions from the QD nanocrystals, producing elemental selenium. Despite the efficient role of the shelling layers toward surface trap passivation, these zinc chalcogenide thin layers are not sufficiently chemically inert to prevent the QD decomposition during in situ radical polymerization. In order to prevent QD oxidation, we explored the role of different ligands that can passivate the QD surface and prevent their decomposition during the polymerization reaction. A screening of QDs aged for 24 h in the presence of a high concentration (0.5 M) of different ligands: oleylamine (OLA), OA, *n*-trioctylphosphine (TOP), and 1-dodecanethiol (DDT) reveals that the thiols are fundamental to guaranteeing the stability of AISe/ZnSe/ZnS QDs during in situ radical polymerization. This can be appreciated by the characteristic photoluminescence of silver indium selenide QDs observed in the composite prepared in the presence of DDT (Figure 6c). It must be remarked that in the presence of the thiol ligand QD stability during the polymerization reaction is ensured, and the QDs can be added at the beginning of the prepolymerization step without any precaution and are stable even in the presence of a relatively high concentration of the initiator (40 mM). A weak blue luminescence can be observed in the sample prepared using OLA and can be related to impurities present in OLA. The role of thiol ligands in QD stabilization can be ascribed to the combination of (i) a strong binding on the QD surface, which successfully reduces the local diffusion of radical species in the proximity of the QD surface, thanks to the steric hindrance of the aliphatic chains, and (ii) their activity as radical quenchers via oxidation to disulfides, which can locally quench the radical species. A similar effect was observed on the stabilization of CdTe QDs by a ligand shell of 3-mercaptopropionic acid during the radical polymerization of OVDAC and polystyrene, and the stability of the luminescence of the nanocrystals was related to the formation of a thick and stable ligand layer.^29^

We further investigate different thiols in terms of their capability of stabilizing the QDs during the polymerization step, comparing 1-dodecanethiol (DDT), *tert*-dodecylmercaptan (TDM), and (3-mercaptopropyl)trimethoxysilane (MPTMS). The UV–vis spectra collected in the transmission mode (Figure S6) show that all the QD/PMMA composites present a distinct shoulder at around 580 nm that is related to the QD absorption. The absorption at longer wavelengths observed for DDT and TDM suggests a higher degree of QD aggregation for these two samples, which causes a loss in the transparency of the composites. This is further supported by the PL characterizations of the composites; the smaller red shift of the QDs emission is observed for MPTMS (10 nm), which shows an emission band centered at 675 nm; instead, DDT and TDM emissions maximums are centered at 695 (30 nm) and 700 (35 nm), respectively.

The AISe/ZnSe/ZnS QDs functionalized with different thiols were investigated to further unravel the role of the interface chemistry in QD stabilization during the polymerization process. FTIR analysis of the QDs after cleaning to remove the excess free ligand is shown in Figure S7. Due to the relatively similar structures, most of the significant bands are observed for both MPTMS, TDM, and DDT. In particular, the bands associated with the aliphatic chains observed around 2920 and 2850 cm^–1^ that can be related to the ν_C–H_ mode in CH_3_ and CH_2_, the sharp band at 1480 cm^–1^ that is associated with δ_CH3_, and the band at 725 cm^–1^ that can be related to the ν_C–S_ characteristic of the thiolate group. The characteristic band at 1080 cm^–1^ observed for MPTMS can be related to the ν_Si-OR_. The optical characterizations for the QDs in solution in the presence of the different thiol ligands are reported in Figure S8. The UV/vis spectra (Figure S8a) show similar features that are characteristic of AISe/ZnSe/ZnS QDs for all the different thiols as well as unpassivated QDs (produced by dispersing the QDs post purification without any further ligand addition).^48^ The PL spectra (Figure S8b) show lower PL intensity for the unpassivated QDs compared to QDs in the presence of the thiols. Moreover, among the different thiols, QDs stabilized in the presence of MPTMS show the largest blue shift (670 nm) along with a significant increase of the PLQY to 65% (with respect to the 46% observed in the presence of DDT), suggesting a most favorable adsorption on the QD surface for MPTMS. The enhancement of the PL intensity can be related to the passivation of the QD surface by thiol ligands, which can prevent nanocrystal aggregation and reduce nonradiative recombination pathways by passivation of surface defects.^49^ QD aggregation induces internal dot energy transfer and promotes emission from larger nanoparticles and nonradiative recombination. It is likely that the superior properties observed for the samples prepared in the presence of MPTMS are due to a most favorable interaction for MPTMS with the surface of the nanocrystals along with the different distribution of steric hindrance in the different thiol molecules.

To further elucidate the role of the interface chemistry on QD stability during in situ radical polymerization, samples of PMMA loaded with AISe/Zn(SeS) core–shell QDs were produced using the same procedure developed for AISe/ZnSe/ZnS QDs (Figure S9a,b). Similar to what was observed for AISe/ZnSe/ZnS QDs, the polymerization conducted in the absence of thiol ligand (pristine AISe/Zn(SeS) QDs) causes the complete loss of luminescence even for AISe/Zn(SeS) QDs that are characterized by a thicker Zn chalcogenide shell. As expected, the addition of MPTMS increases the QD stability during the polymerization step, allowing the production of luminescent AISe/Zn(SeS) QDs PMMA composites. The UV–vis absorption and transmittance spectra (Figure S9c) show broad absorption with a large contribution above 700 nm for the pristine AISe/Zn(SeS) QDs, which is related to the absorption of the selenium microcrystals. Instead, the composites produced using AISe/Zn(SeS) QDs in the presence of MPTMS show the characteristic absorption of the nanocrystals. The transmittance of AISe/Zn(SeS) QDs in the presence of MPTMS at 1000 nm is around 80%; this can be related to the formation of small aggregates. The PL spectra of AISe/Zn(SeS) QDs (Figure S9d) show a significant red shift of the emission to 670 and 650 nm for the composite loaded at 3.0 and 1.5%_w_ respectively, this behavior can be associated with the aggregation of the QDs during polymerization.

**Figure 7:**
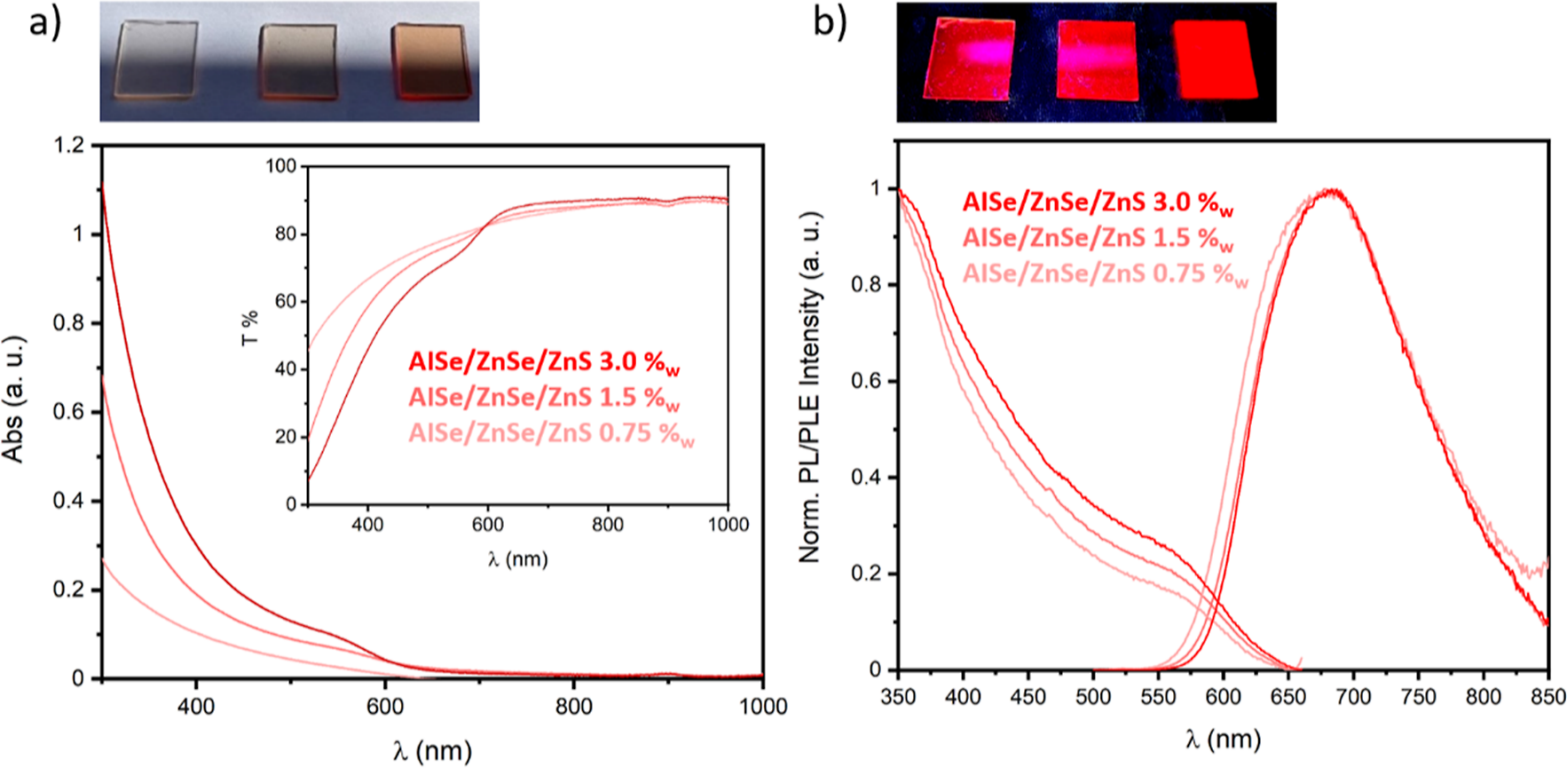
(a) Photograph of the samples under ambient light, UV–vis and transmission spectra of AISe/ZnSe/ZnSe QDs-PMMA. (b) Photograph of the samples under UV light, PLE and PL spectra of AISe/ZnSe/ZnSe QDs-PMMA composites with different QD concentrations.

Thus, for the production of luminescent PMMA composites for the application in red-LED, we used AISe/ZnSe/ZnS QDs stabilized with MPTMS to fabricate PMMA slabs (2.5 cm × 2.5 cm × 0.2 cm) loaded with different nanocrystal amounts at 3, 1.5, and 0.75%_w_ via in situ radical polymerization. The samples show a high level of transparency (Figure S10), and the UV–vis absorption spectra in Figure 7 show the expected band associated with the AISe/ZnSe/ZnS QD absorption at 580 nm, which increases along with the QD concentration in composites. Moreover, low absorption is observed at longer wavelengths, which could be related to QD aggregation. The transmittance spectra Figure 7a show a transmission of 90% around 700 nm for all the composites. In this condition, the QD absorption becomes negligible, and the loss in transmittance of around 10% is commonly related to the reflection from the two surfaces of the slab (2 mm thickness).^26,50^ Interestingly, the transmittance spectra suggest a lower tendency to form aggregates during the polymerization of the smaller AISe/ZnSe/ZnS QDs with respect to the larger AISe/Zn(SeS) QDs. The PL and PLE Figure 7b spectra show an emission peak centered around 677 nm for all the samples and a broad excitation range which covers the whole visible spectrum and starts to decrease around 580 nm. The optical characterizations confirm the high quality of the composites with a low degree of QD aggregation, which is fundamental for several applications in photonics, such as the production of luminescent solar concentrators.^21,22^ Thermogravimetric analysis (TGA) was used to investigate the thermal stability of the composite. The TGA curve of AISe/ZnSe/ZnS QDs-PMMA 1.5%_w_ (Figure S11) shows the typical decomposition profile for PMMA, which starts around 150 °C with a first weight loss that is related to the degradation initiated by radical transfer to the unsaturated chain ends, and the decomposition ends at around 400 °C with the random scission within the polymer chain.^51,52^ Similar observations were reported by other authors on nanocrystals-PMMA composites.^26,30^

**Figure 8:**
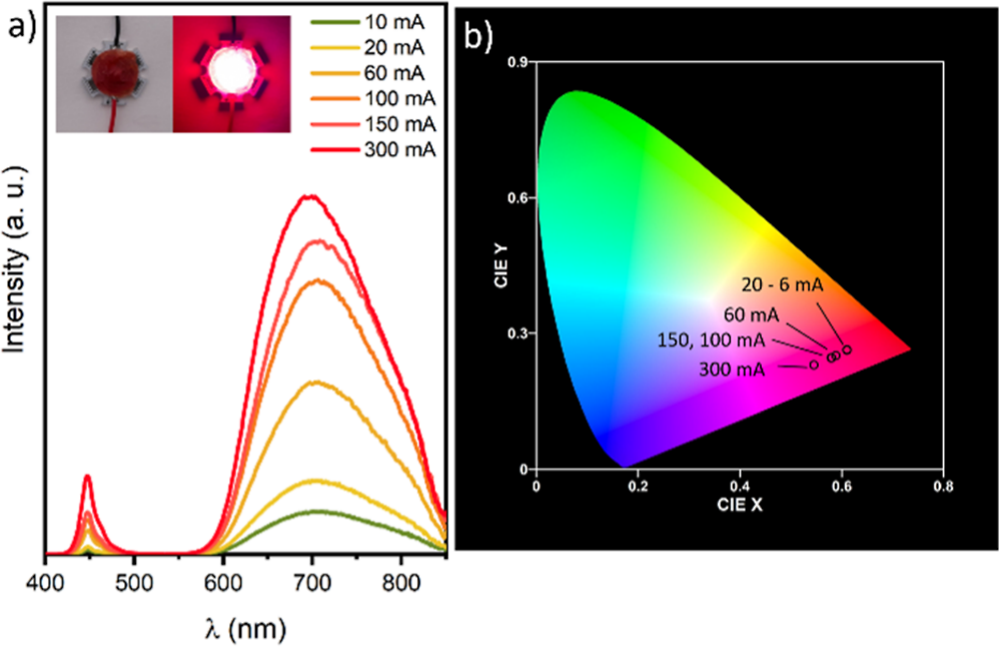
(a) Luminescence spectra and (b) CIE chromaticity coordinate diagram of the AISe/ZnSe/ZnS-PMMA LED device collected at different current intensities. Photograph of the LED device off and on operating at 100 mA (an inset).

To explore the potential application of AISe/ZnSe/ZnS-PMMA in photonics devices, the composite was tested as a color conversion layer for the production of red-LED. Red-LEDs technology is mainly based on AlGaInP; however, a significant reduction in device efficiency is observed in the downscaling of the LED for the production of mini/micro LEDs, and color conversion layers are of large interest for the production of these devices.^53,54^ The most commonly used colorization materials are rare-earth phosphorus, which is affected by several issues, among them the large particle size (1–10 μm) and availability of the precursors.^53,55^ For these reasons, QDs have recently stimulated a large interest as potential candidates for the production of color conversion layers. To produce an AISe/ZnSe/ZnS-PMMA-based red-LED, a 455 nm blue LED (3.0 V) was coated with the layer of AISe/ZnSe/ZnS-PMMA composites. The luminescence spectra of the device (Figure 8a) show a small, sharp contribution from the LED at 455 nm combined with the broad emission originated from the AISe/ZnSe/ZnS QDs. The Commission Internationale d l’Eclairage (CIE) 1931 chromaticity coordinate diagram (Figure 8b) shows that the luminescence spectra are dominated by a deep red color. Increasing the current intensity from 10 mA to 150 mA causes a small variation of the luminescence from (0.61, 0.26) to (0.59, 0.25). More pronounced variations are observed at higher current, such as 300 mA, with a color coordinate of (0.55, 0.23). However, even at a high current, the device luminescence remains in the red range, showing superior performance for the production of red-LEDs with respect to similar systems.^13^

## 3 Conclusions

In summary, an interesting strategy for the production of silver indium selenide QDs shelled with zinc selenide and zinc sulfide layers has been developed. The shelling treatments are fundamental to improving the optical performance of the nanocrystals, increasing the PLQY from 3% as observed for the AISe core to 27 and 46% for the AISe/ZnSe and AISe/ZnSe/ZnS QDs, while maintaining an emission centered in the red spectral range for the multishell system (670 nm). The incorporation of AISe/ZnSe/ZnS QDs in a PMMA matrix via in situ radical polymerization has been investigated for the production of luminescent polymeric composites with potential applications in photonics. The role of proper ligand stabilization has proven to be critical in order to preserve particle stability during the polymerization step. Different thiol ligands have also been explored for the production of highly transparent AISe/ZnSe/ZnS-PMMA slabs with bright red emission. In particular, (3-mercaptopropyl)trimethoxysilane shows the best performance, increasing the PLQY of AISe/ZnSe/ZnS QDs to 65% due to efficient passivation of the surface state as well as the stabilization of nanocrystals against radical-mediated oxidation reactions during the polymerization process. These observations remark the critical role of the interface chemistry for the optimization of the optical properties and chemical stability of silver indium selenide QDs, which are fundamental for the potential application of these systems in photonics devices. Finally, it was demonstrated that the AISe/ZnSe/ZnS-PMMA composites can serve as color conversion layers for the production of red LEDs.

## 4 Methods

### 4.1 Materials

Silver nitrate (AgNO_3_, 99.999%), indium(III) acetate (In(OAc)_3_, 99.99%), selenium powder (Se, 99.99%), sulfur powder (S, ≥99.95%), trioctylphosphine (TOP, 90%), (3-mercaptopropyl)trimethoxysilane (MPTMS, 95%), oleylamine (OLA, ≥98), oleic acid (OA, 90%), 1-octadecane (ODE, 90%), 1-dodecanethiol (DDT, 98%), *tert*-dodecylmercaptan (*t*-DDT, 98.5%), hexane (99%), toluene (99.8%), ethanol (HPLC grade), methanol (HPLC grade), acetone (HPLC grade), chloroform (HPLC grade), lauroyl peroxide (97%), and zinc stearate (90%). All reagents used in this experiment were purchased from Sigma-Aldrich (Merck) and used without further purification. Methyl methacrylate (MMA, 99%) was purchased from Sigma-Aldrich (Merk) and purified by vacuum distillation before use.

### 4.2 Characterization

UV–vis absorption spectra were collected with an Agilent Technologies Cary 60 UV–vis spectrophotometer using a 1.0 cm quartz cuvette. TEM and HR-TEM were performed using a Jeol 2100 microscope working at 200 kV. X-ray diffraction (XRD) was used to determine the sample’s structure. XRD data were obtained using a Bruker D2 Phaser X-ray Powder Diffractometer working with the Cu Kα (λ = 1.54184) radiation source. Energy-dispersive X-ray spectroscopy (EDS) was employed to characterize the sample’s composition. The analysis was performed using a Zeiss Ultra plus SEM equipped with an Oxford Instruments 80 mm^2^ XMAX EDX detector working with an acceleration voltage of 20 keV. Photoluminescence excitation and emission spectra of QD solutions were measured using a Horiba FluoroMax 4 Spectrofluorometer working with 2.0 nm of excitation and emission slits. The photoluminescence decay analyses were performed using a Horiba Fluorolog spectrophotometer working with a NanoLed 458 nm excitation source. PLQYs were determined by comparing the area of photoluminescence emission spectra of QDs in toluene with the rhodamine 6G standard in water (PLQY = 92%). For the measurements, an excitation light at 490 nm was employed. The PLQY of the QD solution is calculated according to eqs S3 and S4. PerkinElmer Pyris 1 TGA was used for the TGA, working with a 30 to 900 °C, 10 °C/min ramp under a nitrogen atmosphere.

### 4.3 AISe QD Synthesis

Silver nitrate (0.2 mmol) and indium acetate (0.6 mmol) were dispersed in ODE (10.0 mL), OA (2.0 mL), and DDT (4.0 mL). The mixture was degassed at 80 °C under vacuum for 30 min. Subsequently, the atmosphere was switched to argon, and the temperature was raised to 140 °C. Meanwhile, another flask was used for the preparation of the Se precursor by solubilizing selenium powder (1.1 mmol) in OLA (4.0 mL) and DDT (270 μL). The selenium precursor solution was rapidly injected at 140 °C. Then, the reaction mixture was heated at 140 °C for 30 min, after which the reaction was quenched in cold water. The product was transferred to a centrifuge tube, precipitated with methanol and ethanol, and then collected by centrifugation at 9000 rpm for 2 min. The pellet was dispersed in toluene. This purification step was repeated three times, and the purified product was dispersed in 20.0 mL of toluene for further use.

### 4.4 AISe/ZnS QD Synthesis

3.0 mL of the AISe QD core solution was added to OA (2.0 mL), OAm (2.0 mL), DDT (6.0 mL), and ODE (6.0 mL). The solution was degassed at 80 °C for 30 min under vacuum, and then, the flask was filled with argon and the temperature was raised to 130 °C. The ZnSe precursor solution was prepared by solubilizing selenium powder (0.4 mmol) and Zn(OAc)_2_ (0.4 mmol) in TOP (3.0 mL) and OLA (200 μL). The solution was degassed at 80 °C for 30 min prior to the injection. When the temperature of the reaction mixture reached 130 °C, 3.0 mL of the ZnSe precursor was slowly injected over 30 min. After the injection, the mixture was maintained at 130 °C for another 30 min. When the reaction was finished, the reaction mixture was cooled in a water bath. The product was transferred to a centrifuge tube, precipitated with methanol and ethanol, and then collected by centrifugation at 9000 rpm for 2 min. The pellet was dispersed in toluene. This purification step was repeated three times. The purified AISe/ZnSe QDs were then dispersed in a 3.0 mL toluene solution for further use.

### 4.5 AISe/ZnSe/ZnS QD Synthesis

3.0 mL of the AISe/ZnSe QD solution was added to OA (2.0 mL), OAm (2.0 mL), DDT (6.0 mL), and ODE (6.0 mL). The solution was degassed at 80 °C for 30 min under vacuum, and then, the flask was filled with argon, and the temperature was raised to 130 °C. The ZnS precursor solution was prepared by solubilizing sulfur powder (0.4 mmol) and Zn(OAc)_2_ (0.4 mmol) in TOP (3.0 mL) and OLA (200 μL). The solution was degassed at 80 °C for 30 min prior to injection. When the temperature of the reaction mixture reached 130 °C, 3.0 mL of the ZnS precursor was slowly injected over 30 min. After injection, the mixture was maintained at 130 °C for another 30 min. When the reaction finished, the flask was immediately cooled in cold water bath. The product was transferred to a centrifuge tube, precipitated with methanol and ethanol, and then collected by centrifugation at 9000 rpm for 2 min. The pellet was dispersed in toluene. This purification step was repeated three times. The purified AISe/ZnSe/ZnS QDs were then dispersed in a 3.0 mL toluene solution for further use.

### 4.6 PMMA/QD Composite Preparation

A solution of MPTMS (0.5 M) and AISe/ZnSe/ZnS QDs (10 mg/mL) was prepared in toluene, and the QDs were aged for 24 h at 4 °C in the presence of the ligand prior to their use. Then, different amounts of QD solution were added to methyl methacrylate (6.0 mL) and lauroyl peroxide (0.1 mmol). The solution was heated at 60 °C for 2 h under an argon atmosphere to obtain a dense syrup. The syrup was then transferred to a casting mold made of two temperate glass slabs separated by a PVC gasket. The mold was placed in an oven at 70 °C for 24 h. After cooling, the PMMA slab with the desired size was cut, and the fresh edges were polished.

### 4.7 Fabrication of the AISe/ZnSe/ZnS QD-Based LED

AISe/ZnSe/ZnS QDs-PMMA syrup was produced as reported above, with the only difference being that the prepolymerization was continued for 3 h instead of 2 h to produce a more viscous syrup. Then, the syrup was deposited on top of a 3.0 V, 455 nm blue LED chip. The dense syrup was polymerized in an oven at 50 °C overnight and finally polished. The luminescence spectra of the device were collected using a Horiba Fluoromax 4 spectrophotometer equipped with an integrating sphere and working with 2 nm slits in the emission path.

## Supporting Information Available

EDS spectra; Tauc plot; lifetime decay data and data analysis of QD colloidal solutions; determination of the PLQY; UV–vis absorption and PL spectra of QD/PMMA composites; and TGA data (PDF)

## Abbreviations

QDs: quantum dots
PMMA: poly(methyl methacrylate)
LED: light emitting diode
PLQY: photoluminescence quantum yield
OLA: oleylamine
OA: oleic acid
TOP: *n*-trioctylphosphine
DDT: 1-dodecanethiol
TDM: *tert*-dodecylmercaptan
MPTMS: (3-mercaptopropyl)trimethoxysilane
