# Supplementary material for: Multishell Silver Indium Selenide-Based Quantum Dots and Their Poly(methyl methacrylate) Composites for Application in Red-Light-Emitting Diodes
Source: ACS Appl Mater Interfaces. 2024 Jul 5;16(28):37017–27. doi: 10.1021/acsami.4c06433 (PMC11261562; doi:10.1021/acsami.4c06433)
Supplement: Supplementary file 1 — am4c06433_si_001.pdf [file am4c06433_si_001.pdf]

Electronic Supplementary information

Multi-shell silver indium selenide based quantum dots and their polymethylmethacrylate composites for application in red-light emitting diodes

Lorenzo Branzi, Jinming Liang, Garret Dee, Aoife Kavanagh, Yurii K. Gun'ko**

School of Chemistry, CRANN and AMBER Research Centres, Trinity College Dublin, College Green, Dublin 2, D02 PN40, Ireland

branzil@tcd.ie, igounko@tcd.ie

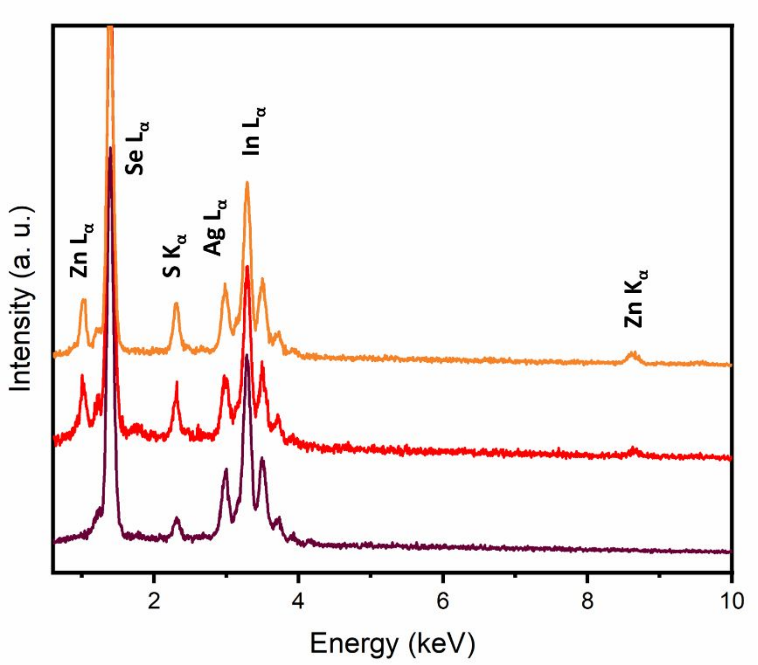

Figure S1: EDS spectra of AISe (brown), AISe/ZnSe (red) and AISe/ZnSe/ZnS (orange) QDs.

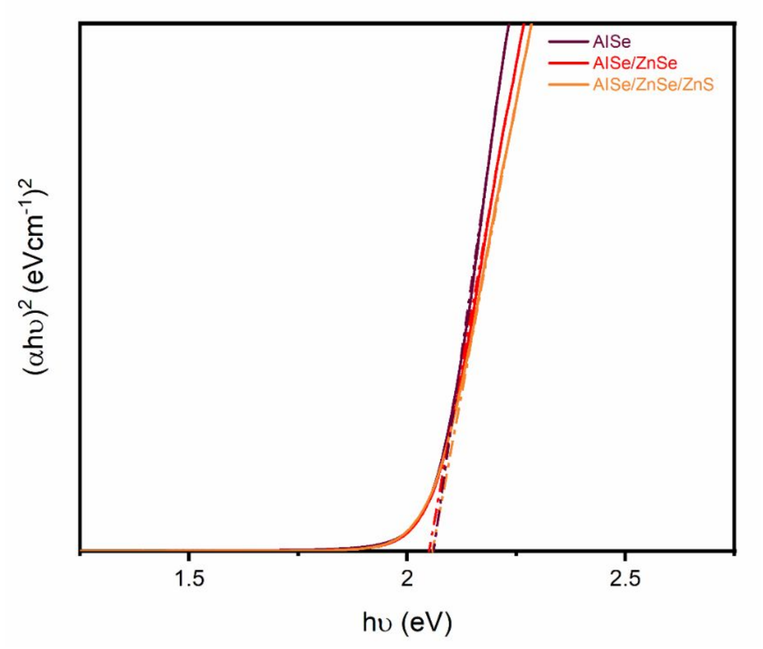

Figure S2: Tauc plot of AISe (brown), AISe/ZnSe (red) and AISe/ZnSe/ZnS (orange) QDs.

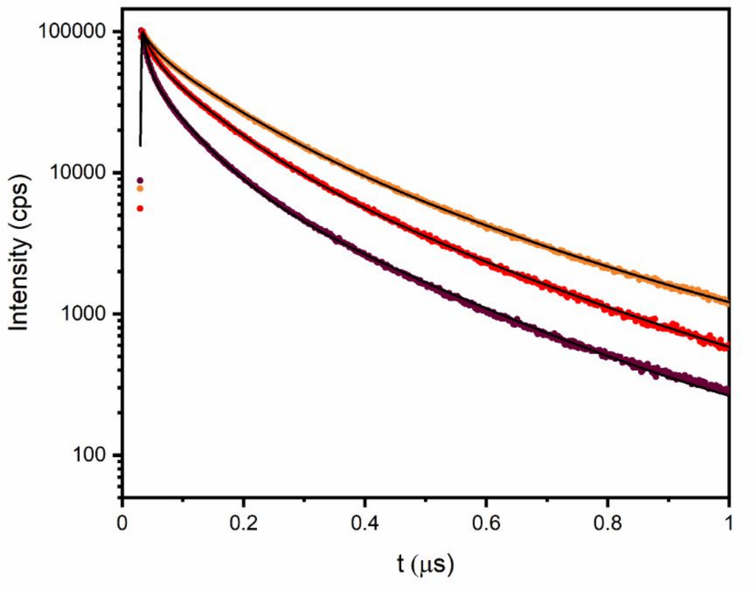

Figure S3: photoluminescence decay curve and triexponential fitting of AISe (brown), AISe/ZnSe (red) and AISe/ZnSe/ZnS (orange) QDs.

Equation S1

$$I(t) = A_1 e^{-\frac{t}{\tau_1}} + A_2 e^{-\frac{t}{\tau_2}} + A_3 e^{-\frac{t}{\tau_3}}$$

Equation S2

$$\tau_{Av} = \frac{\sum A_i \tau_i^2}{\Sigma A_i \tau_i}$$

Table S1: results of the analysis of the decay data

	τ_1 (ns)	A_1 (a.u.)	τ_2 (ns)	A_2 (a.u.)	τ_3 (ns)	A_3 (a.u.)	τ_{av} (ns)
AISe	63.9	0.475	224.8	0.456	8.63	0.0961	187
AISe/ZnSe	95.4	0.478	175.8	0.062	265	0.460	216
AISe/ZnSe/ZnS	113.9	0.452	20.55	0.035	299.5	0.512	252

Equation S3[1,2]

$$\Phi_{QDs} = \Phi_{st} \frac{I_{QDs} f_{st} n_{QDs}^2}{I_{st} f_{QDs} n_{st}^2}$$

Where, Φ represents the photoluminescent quantum yield, I is the integrated intensity of the photoluminescent spectra, f is the absorption factor at the excitation wavelength and n is the diffraction index of the solvent. Finally, the subscripts QDs and st refer to the sample and the standard respectively.

Equation S4[1,2]

$$f_{QDs,st} = 1 - 10^{\wedge}(-A_{QDs,\,st})$$

Where *f* and *A* represent the absorption factor and the absorbance for quantum dots (*QDs*) and fluorescence standard (*st*).

Thick shell AlSe/Zn(SeS) QDs, synthesis and characterisations: thick shell AlSe/Zn(SeS) are produced using the synthetic scheme represented in (**Figure S4**). In this approach, a dispersion of zinc stearate in the presence of selenium and 1-dodecanthiol as the selenium and sulfur sources respectively is slowly injected into the reaction mixture containing AlSe QDs core at 180 °C.

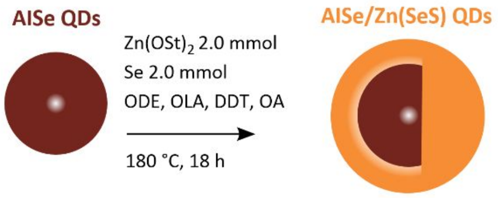

Figure S4: representation of the synthetic scheme used for the production of AlSe/Zn(SeS) QDs.

Synthesis: 12.0 ml of reaction mixture containing AlSe QDs was degassed at 80 °C for 30 minutes to remove any volatile species formed during the synthesis. The temperature was raised to 180 °C and a mixture of zinc stearate (2.0 mmol) and selenium (2.0 mmol) dispersed in 1-octadecene (10.0 ml), 1-oleylamine (4.0 ml), 1-dodecanthiol (1.0 ml) and oleic acid (5.0 ml) was injected at the speed of 2.0 ml/h for 10 h. Then, the reaction mixture was maintained at 180 °C for further 8 h after the injection. When the reaction finished, the reaction mixture was cooled in a water bath. The product was transferred to a centrifuge tube, precipitated with methanol and ethanol, and then collected by centrifugation at 9000 rpm for 5 minutes.

The pellet was dispersed in toluene. This purification step was repeated three times. The purified AISe/Zn(SeS) QDs were then dispersed in a 5.0 ml toluene solution for further use.

Characterisations: the addition of the zinc chalcogenide precursor solution causes the gradual blue shift of the UV-Vis absorption (**Figure S5a**) as expected for the diffusion of zinc in the QDs core, inducing the increasing of the band gap.[3,4] Similar behavior is observed in the PL spectra (**Figure S5b**), the emission peak shifts with the increasing addition of the zinc chalcogenides precursor from 660 nm observed after the first hour of addition to 635, 628 and finally 610 nm for 3, 6 and 18 h respectively. A significant reduction of the FWHM from 141 to 122 nm and an increasing of the PLQY from 3 to 53% is observed. TEM (**Figure S5c**) shows the increasing of the nanocrystal size from 2.4 ± 0.5 nm to 4.0 ± 0.7 nm (**Figure S5d**) due to the deposition of the zinc chalcogenide shell. The XRD pattern (**Figure S5e**) shows a shifting of the diffraction of AISe QDs chalcopyrite core to higher angles in line with the deposition of the zinc chalcogenide shell.[5] In particular, the (112) diffraction is observed at 26.7 °2θ, while the diffraction from the (201, 220) and (312,116) planes are observed at 44.6 and 52.7 °2θ respectively. Moreover, the diffractions at 64.2 and 71.5 °2θ can be related to (400) and (331) planes of sphalerite phase of ZnSe.[4,6] The EDS analysis (**Figure S5f)** confirms that the final AISe/ZnSeS QDs retain the original silver to indium ratio of 1:2:91. Moreover, a

sulfur to selenium ratio of 1:1.8 and silver to zinc ratio of 1:6.8 is observed, considerably higher than that observed for the AISe/ZnSe/ZnS QDs sample characterised by thin shells.

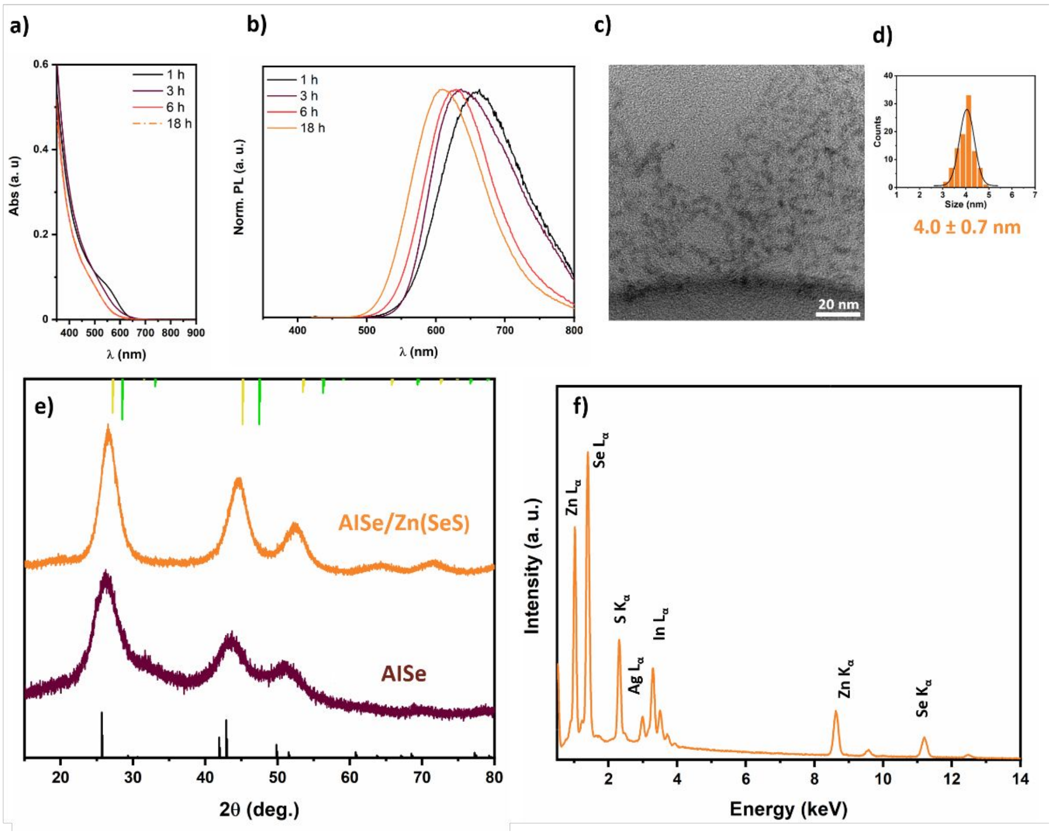

Figure S5: UV/Vis absorption a) and PL b) spectra of AISe/Zn(SeS) QDs at different stages of the zinc chalcogenide precursor addition. TEM micrograph c) and size distribution histogram d) of AISe/Zn(SeS) QDs. e) XRD pattern of AISe core and AISe/Zn(SeS) core shell systems. Reference pattern: $AgInSe_2$ chalcopyrite phase ICSD28751 (black), ZnSe sphalerite phase ICSD77091 (yellow) and ZnS sphalerite phase ICSD77090 (green). f) EDS spectrum of AISe/Zn(SeS) QDs.

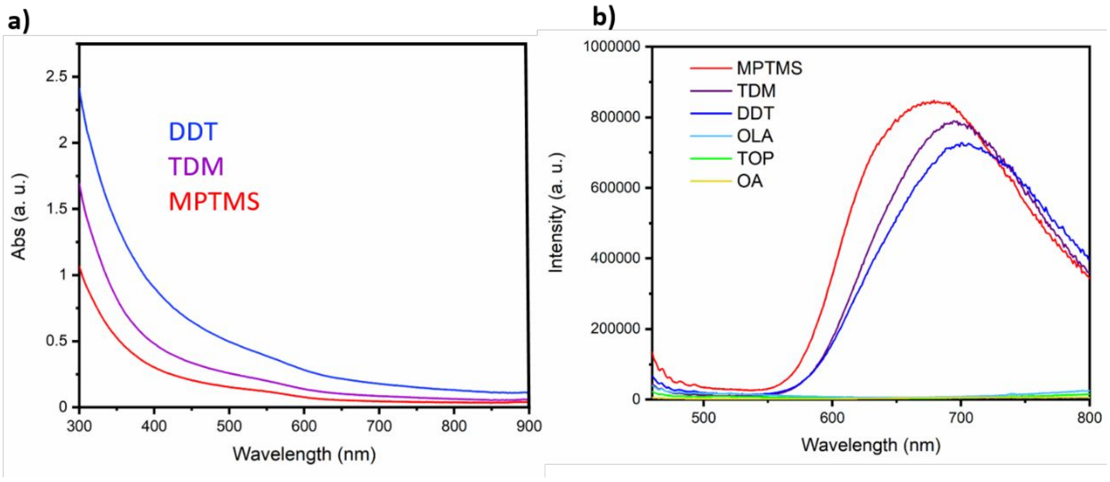

Figure S6: UV-Vis absorption (a) and photoluminescence (b) spectra of AISe/ZnSe/ZnS-PMMA composites prepared in the presence of different thiol ligands: DDT (blue), TDM (purple), MPTMS (red), OLA (light blue), TOP (green) and OA (yellow).

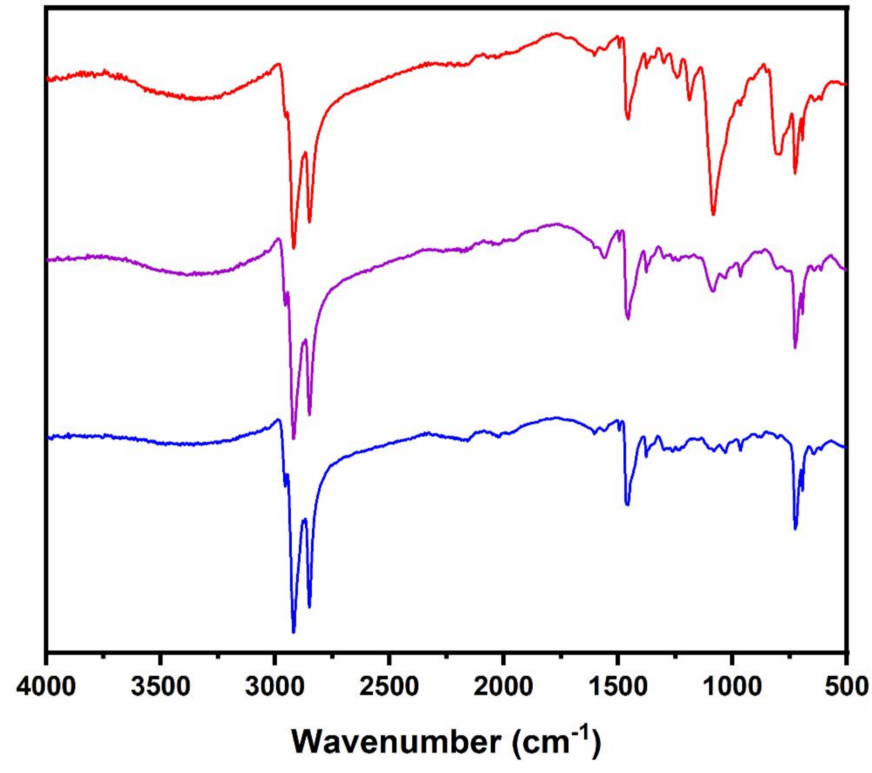

Figure S7: FTIR analysis of AISe/ZnSe/ZnS QDs after passivation with MPTMS (red), TMD (purple) and DDT (blue).

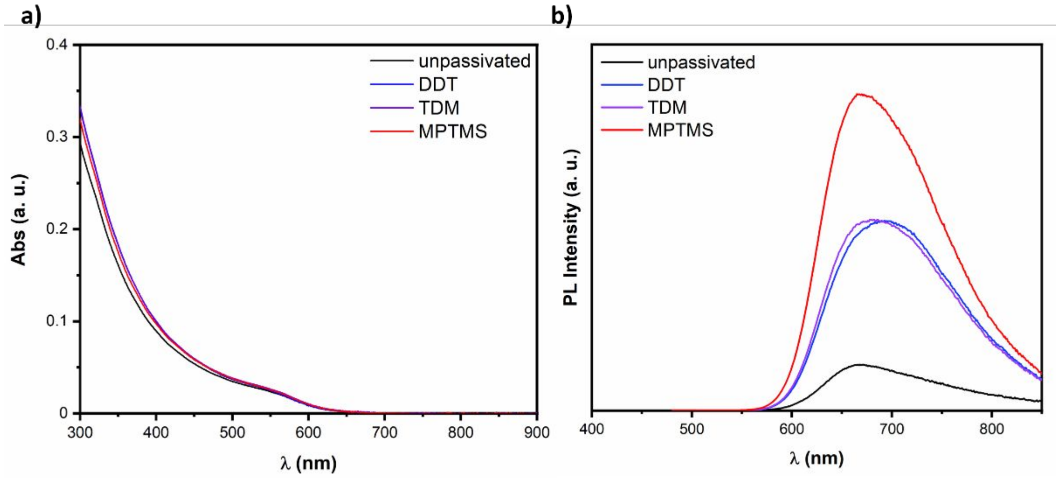

Figure S8: a) UV-Vis absorption and b) PL spectra of AISe/ZnSe/ZnS QDs un-passivated (dispersed without addition of further ligand after cleaning) and passivated by different thiol ligands in toluene.

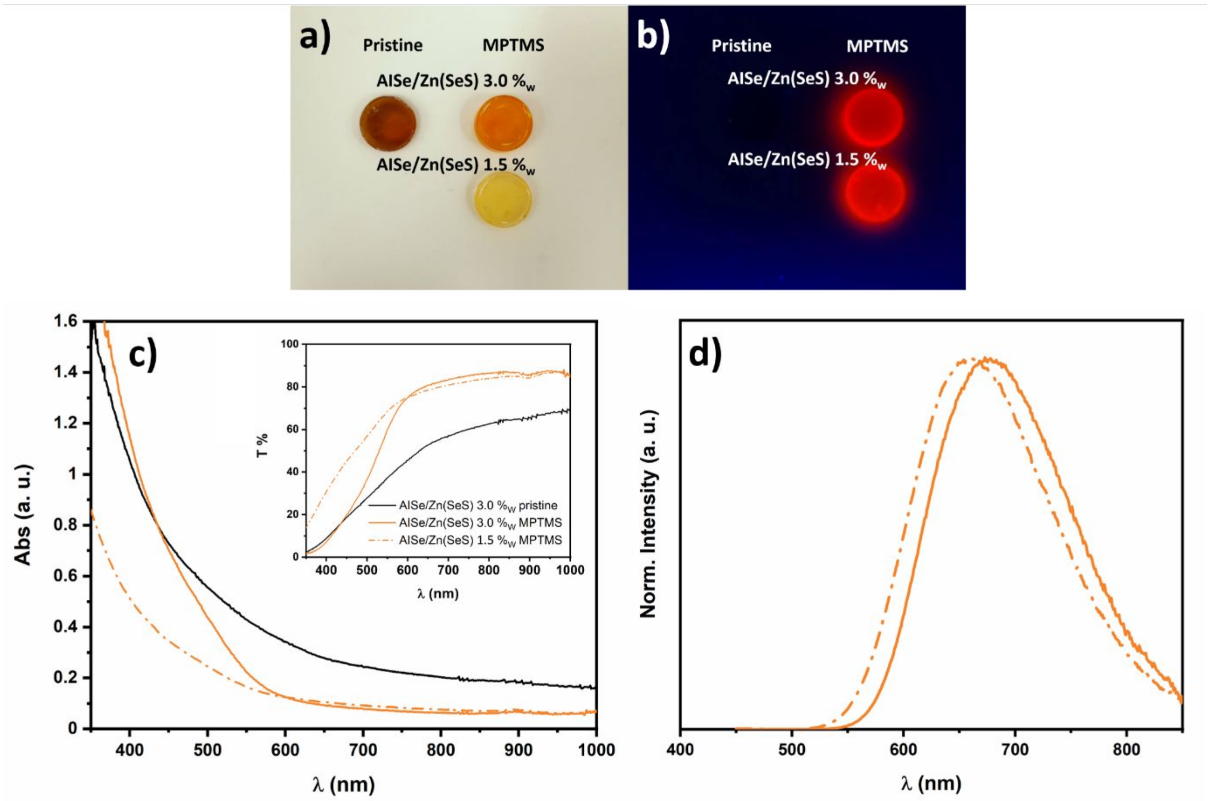

Figure S9: Photographs of AISe/Zn(SeS) PMMA composites produced without (pristine) and in the presence of MPTMS under ambient a) and UV b) light. c) UV-Vis absorption and transmittance spectra of AISe/Zn(SeS) PMMA composites. d) PL spectra of AISe/Zn(SeS) 3.0%$_w$ (solid line) and AISe/Zn(SeS) 1.5%$_w$ (dashed line) PMMA composite produced in the presence of MPTMS.

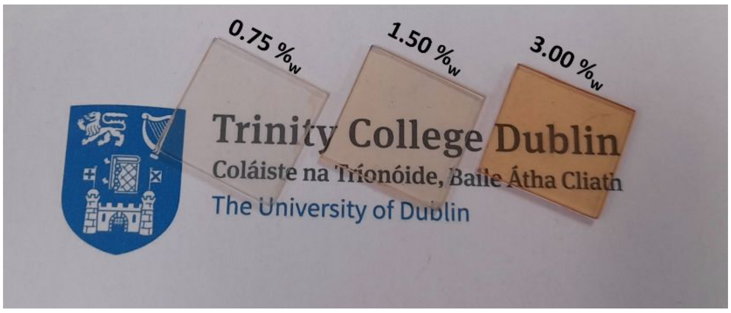

Figure S10: photographs of AlSe/ZnSe/ZnS -PMMA composites with different QDs amounts.

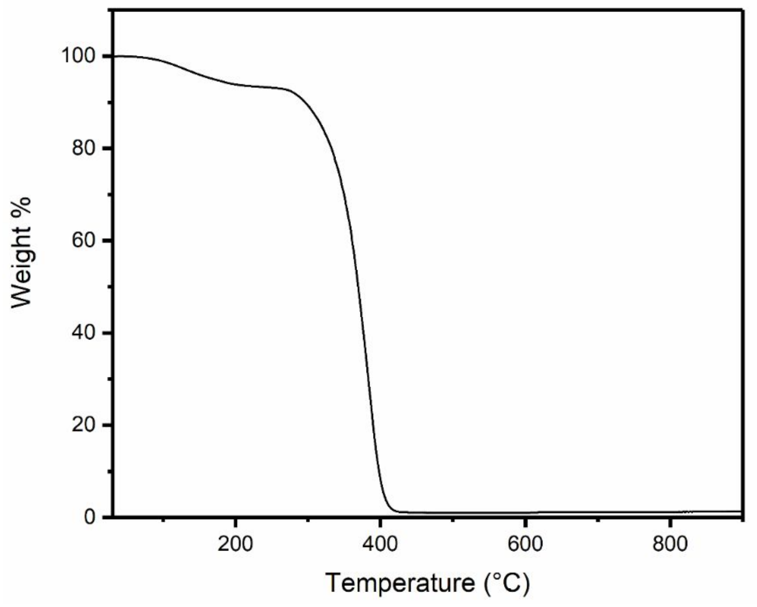

Figure S11: TGA analysis of AlSe/ZnSe/ZnS-PMMA 1.5%$_w$ composites.

REFERENCES:

1 U. Resch-Genger and K. Rurack, *Pure Appl. Chem.*, 2013, **85**, 2005–2026.

2 A. M. Brouwer, *Pure Appl. Chem.*, 2011, 83, 2213–2228.

3 J. Park and S. W. Kim, *J. Mater. Chem.*, 2011, **21**, 3745–3750.

4 D. Voigt, M. Bredol and A. Gonabadi, *Opt. Mater. (Amst).*, 2021, **115**, 110994.

5 P. M. Allen and M. G. Bawendi, *J. Am. Chem. Soc.*, 2008, **130**, 9240–9241.

6 A. Zhang, C. Dong, L. Li, J. Yin, H. Liu, X. Huang and J. Ren, *Sci. Rep.*, 2015, **5**, 1–13.
